# Supplementary material for: COZOID: contact zone identifier for visual analysis of protein-protein interactions
Source: BMC Bioinformatics. 2018 Apr 6;19:125. doi: 10.1186/s12859-018-2113-6 (PMC5889581; doi:10.1186/s12859-018-2113-6)
Supplement: Supplementary file 2 — Software build. Executable binary file of the software tool. (ZIP 118,784 kb) [file 12859_2018_2113_MOESM2_ESM.zip › cozoid/java/docs/beansbinding-1.2.1-doc.zip!/index-files/index-12.html]

L-Index (Beans Binding)


---


|  |  |  |
| --- | --- | --- |
| **Overview** \| Package \| Class \| Use \| **Tree** \| **Deprecated** \| **Index** \| **Help** | |  |
| **PREV LETTER**   **NEXT LETTER** | **FRAMES**    **NO FRAMES**     **All Classes** |  |


A B C D E F G H I J K L M N O P Q R S T U V W 

---


## **L**

**LBRACK** - Static variable in interface org.jdesktop.el.impl.parser.ELParserConstants: **LE0** - Static variable in interface org.jdesktop.el.impl.parser.ELParserConstants: **LE1** - Static variable in interface org.jdesktop.el.impl.parser.ELParserConstants: **LETTER** - Static variable in interface org.jdesktop.el.impl.parser.ELParserConstants: **LexicalError(boolean, int, int, int, String, char)** - Static method in error org.jdesktop.el.impl.parser.TokenMgrError: Returns a detailed message for the Error when it is thrown by the token manager to indicate a lexical error. **lexStateNames** - Static variable in class org.jdesktop.el.impl.parser.ELParserTokenManager: **line** - Variable in class org.jdesktop.el.impl.parser.SimpleCharStream: **ListBindingManager** - Class in org.jdesktop.swingbinding.impl: **ListBindingManager()** - Constructor for class org.jdesktop.swingbinding.impl.ListBindingManager: **listElementPropertyChanged(ObservableList, int)** - Method in interface org.jdesktop.observablecollections.ObservableListListener: Notification than a property of an element in this list has changed. **listElementPropertyChanged(ObservableList, int)** - Method in class org.jdesktop.swingbinding.impl.ListBindingManager: **listElementReplaced(ObservableList, int, Object)** - Method in interface org.jdesktop.observablecollections.ObservableListListener: Notification that an element has been replaced by another in the list. **listElementReplaced(ObservableList, int, Object)** - Method in class org.jdesktop.swingbinding.impl.ListBindingManager: **listElementsAdded(ObservableList, int, int)** - Method in interface org.jdesktop.observablecollections.ObservableListListener: Notification that elements have been added to the list. **listElementsAdded(ObservableList, int, int)** - Method in class org.jdesktop.swingbinding.impl.ListBindingManager: **listElementsRemoved(ObservableList, int, List)** - Method in interface org.jdesktop.observablecollections.ObservableListListener: Notification that elements have been removed from the list. **listElementsRemoved(ObservableList, int, List)** - Method in class org.jdesktop.swingbinding.impl.ListBindingManager: **ListELResolver** - Class in org.jdesktop.el: Defines property resolution behavior on instances of `List`. **ListELResolver()** - Constructor for class org.jdesktop.el.ListELResolver: Creates a new read/write `ListELResolver`. **ListELResolver(boolean)** - Constructor for class org.jdesktop.el.ListELResolver: Creates a new `ListELResolver` whose read-only status is determined by the given parameter. **listeningStarted(S)** - Method in class org.jdesktop.beansbinding.BeanProperty: **listeningStarted(S)** - Method in class org.jdesktop.beansbinding.ELProperty: **listeningStarted(S)** - Method in class org.jdesktop.beansbinding.PropertyHelper: Called when this `PropertyHelper` changes from having no listeners installed for the given source object to having listeners installed for the given source object. **listeningStarted()** - Method in class org.jdesktop.swingbinding.adapters.AbstractButtonAdapterProvider.Adapter: **listeningStarted()** - Method in class org.jdesktop.swingbinding.adapters.BeanAdapterBase: **listeningStarted()** - Method in class org.jdesktop.swingbinding.adapters.JComboBoxAdapterProvider.Adapter: **listeningStarted()** - Method in class org.jdesktop.swingbinding.adapters.JListAdapterProvider.Adapter: **listeningStarted()** - Method in class org.jdesktop.swingbinding.adapters.JSliderAdapterProvider.Adapter: **listeningStarted()** - Method in class org.jdesktop.swingbinding.adapters.JSpinnerAdapterProvider.Adapter: **listeningStarted()** - Method in class org.jdesktop.swingbinding.adapters.JTableAdapterProvider.Adapter: **listeningStarted()** - Method in class org.jdesktop.swingbinding.adapters.JTextComponentAdapterProvider.Adapter: **listeningStopped(S)** - Method in class org.jdesktop.beansbinding.BeanProperty: **listeningStopped(S)** - Method in class org.jdesktop.beansbinding.ELProperty: **listeningStopped(S)** - Method in class org.jdesktop.beansbinding.PropertyHelper: Called when this `PropertyHelper` changes from having listeners installed for the given source object to having no listeners installed for the given source object. **listeningStopped()** - Method in class org.jdesktop.swingbinding.adapters.AbstractButtonAdapterProvider.Adapter: **listeningStopped()** - Method in class org.jdesktop.swingbinding.adapters.BeanAdapterBase: **listeningStopped()** - Method in class org.jdesktop.swingbinding.adapters.JComboBoxAdapterProvider.Adapter: **listeningStopped()** - Method in class org.jdesktop.swingbinding.adapters.JListAdapterProvider.Adapter: **listeningStopped()** - Method in class org.jdesktop.swingbinding.adapters.JSliderAdapterProvider.Adapter: **listeningStopped()** - Method in class org.jdesktop.swingbinding.adapters.JSpinnerAdapterProvider.Adapter: **listeningStopped()** - Method in class org.jdesktop.swingbinding.adapters.JTableAdapterProvider.Adapter: **listeningStopped()** - Method in class org.jdesktop.swingbinding.adapters.JTextComponentAdapterProvider.Adapter: **Literal()** - Method in class org.jdesktop.el.impl.parser.ELParser: **LITERAL\_EXPRESSION** - Static variable in interface org.jdesktop.el.impl.parser.ELParserConstants: **LiteralExpression()** - Method in class org.jdesktop.el.impl.parser.ELParser: **localName** - Variable in class org.jdesktop.el.impl.lang.FunctionMapperImpl.Function: **localName** - Variable in class org.jdesktop.el.impl.parser.AstFunction: **LONG** - Static variable in class org.jdesktop.el.impl.lang.ELArithmetic: **lookingAhead** - Variable in class org.jdesktop.el.impl.parser.ELParser: **LPAREN** - Static variable in interface org.jdesktop.el.impl.parser.ELParserConstants: **LT0** - Static variable in interface org.jdesktop.el.impl.parser.ELParserConstants: **LT1** - Static variable in interface org.jdesktop.el.impl.parser.ELParserConstants

---


|  |  |  |
| --- | --- | --- |
| **Overview** \| Package \| Class \| Use \| **Tree** \| **Deprecated** \| **Index** \| **Help** | |  |
| **PREV LETTER**   **NEXT LETTER** | **FRAMES**    **NO FRAMES**     **All Classes** |  |


A B C D E F G H I J K L M N O P Q R S T U V W 

---
